# 1048. Double-Blind, Randomized, Placebo-Controlled Phase 2b Multicenter Trial of V160, a Replication-Defective Human Cytomegalovirus (CMV) Vaccine

**DOI:** 10.1093/ofid/ofab466.1242

**Published:** 2021-12-04

**Authors:** Rituparna Das, Daniel Blazquez-Gamero, David I Bernstein, Soren Gantt, Oliver Bautista, Karen Beck, Anthony Conlon, Daniel Rosenbloom, Dai Wang, Michael Ritter, Beth Arnold, Paula Annunziato, Kevin Russell

**Affiliations:** 1 Merck & Co., Inc., Kenilworth, New Jersey; 2 Pediatric Infectious Diseases Unit, Hospital Universitario; 3 de Octubre and Universidad Complutense, Instituto de Investigación Hospital; 4 de Octubre (imas12) RECLIP, Madrid, Madrid, Spain; 5 Cincinnati Children’s Hospital Medical Center, University of Cincinnati, Cincinnati, OH; 6 CHU Sainte-Justine Research Centre, Université de Montréal, Montreal, Quebec, Canada; 7 Merck and Co Inc., North Wales, PA

## Abstract

**Background:**

Preventing congenital cytomegalovirus infection (CMVi) is an important unmet need. Natural maternal immunity to CMV acquired prior to pregnancy appears to reduce fetal transmission. In a Phase 1 trial, V160, a replication-defective CMV vaccine expressing the pentameric complex, induced humoral and cell-mediated immune (CMI) responses comparable to natural immunity.

**Methods:**

Healthy, CMV-seronegative women aged 16–35 years were randomized 1:1:1 to receive double-blind V160 in a 3- or 2-dose regimen or placebo. Primary and secondary endpoints were efficacy in reducing the incidence of CMVi with 3-dose or 2-dose regimens of V160 vs placebo, respectively, using a fixed-event design. Monthly urine and saliva samples were collected to identify CMVi by polymerase chain reaction (PCR) with a single positive sample considered evidence of infection. Immunoglobulin G (IgG) binding to glycoprotein B (gB) and CMV-specific neutralizing antibody (NAb) were measured in all participants, and CMI responses were measured in a subset. Injection-site and systemic adverse events (AEs) were collected for 5 days and 14 days, respectively, after each vaccination and serious AEs were collected for the trial duration.

**Results:**

2200 women from 7 countries were enrolled (of 7458 screened). Over 80% of participants received all doses, and compliance with saliva and urine samples was > 95%. Vaccine efficacy (VE) of 42.4% (95% CI -13.5, 71.1%) was demonstrated in the 3-dose group vs placebo. In the 2-dose group, VE was -32.0% (95% CI -135.0, 25.0%). Both the quantity and duration of CMV shedding in urine and saliva among cases of CMVi decreased in the 3-dose, but not the 2-dose group vs placebo. Both V160 regimens elicited humoral and CMI responses detected by CMV-specific NAb, gB IgG, and ELISpot, which peaked at Month 7 and continued to be detectable at Month 24. Mild to moderate AEs were more frequently reported in V160 vs placebo recipients, but no vaccine-related serious AEs or deaths were reported.

**Conclusion:**

V160 was well tolerated and immunogenic, but neither the 3-dose nor 2-dose regimen demonstrated significant efficacy against CMVi as defined in this trial. The quantity and duration of CMV shedding was reduced in the 3-dose group, suggesting V160 may improve immune control of viral replication after CMVi.

**Disclosures:**

**Rituparna Das, MD**, **Merck & Co, Inc.** (Employee) **Daniel Blazquez-Gamero, MD**, **MSD** (Other Financial or Material Support, Fees for lectures in educational activities) **Soren Gantt, MD**, **Altona Diagnostics** (Research Grant or Support)**Merck** (Consultant, Grant/Research Support)**Meridian Biosciences** (Research Grant or Support)**Moderna** (Consultant, Research Grant or Support)**VBI Vaccines Inc** (Research Grant or Support) **Oliver Bautista, PhD**, **Merck & Co, Inc.** (Employee) **Karen Beck, RN, BSN**, **Merck & Co, Inc.** (Employee) **Anthony Conlon, PhD**, **Merck & Co, Inc.** (Employee) **Daniel Rosenbloom, PhD**, **Merck & Co, Inc.** (Employee) **Dai Wang, PhD**, **Merck & Co, Inc.** (Employee) **Michael Ritter, BA**, **Merck & Co, Inc.** (Employee) **Beth Arnold, MS**, **Merck & Co, Inc.** (Employee, Shareholder) **Paula Annunziato, MD**, **Merck & Co, Inc.** (Employee) **Kevin Russell, MD, MTM&H**, **Merck & Co., Inc.** (Employee, Shareholder)

